# Supplementary material for: Emissions of Fungal Volatile Organic Compounds in Residential Environments and Temporal Emission Patterns: Implications for Sampling Methods
Source: Int J Environ Res Public Health. 2022 Oct 2;19(19):12601. doi: 10.3390/ijerph191912601 (PMC9564960; doi:10.3390/ijerph191912601)
Supplement: Supplementary file 1 [file ijerph-19-12601-s001.zip › mVOCs emission_SI.pdf]

Supplementary Material

Emissions of Fungal Volatile Organic Compounds in Residential Environments and Temporal Emission Patterns: Implications for Sampling Methods

Kyunghoon Kim, Suyeon Lee, Yelim Choi, Daekeun Kim

Department of Environmental Engineering, Seoul National University of Science and Technology, Seoul 01811, Republic of Korea

S1. Determination of fungal concentrations

S2. Analysis of samples obtained from active air samplers

S3. Analysis of samples obtained from passive air samplers

Table S1. Characteristics of 11 residential environments recruited for our field experiments.

Table S2. Description of analysis conditions of thermal desorption coupled with gas chromatography/mass spectrometry (TD-GC-MS).

Table S3. Detections of compounds from *Aspergillus niger* and the summary statistics in their emission (Microsoft Excel)

Table S4. Detections of compounds from *Alternaria alternata* and the summary statistics in their emission (Microsoft Excel)

Table S5. Detections of compounds from *Penicillium chrysogenum* and the summary statistics in their emission (Microsoft Excel)

Table S6. Numbering of compounds in clustering analysis results for silicone rubber. (Microsoft Excel)

Figure S1. Schematic drawing of experimental setup for mVOCs emission testing.

Figure S2. Temporal emission patterns of mVOCs originating from *Aaspergillus niger* grown on MEA.

Figure S3. Temporal emission patterns of mVOCs originating from *Alternaria alternata* grown on MEA.

Figure S4. Temporal emission patterns of mVOCs originating from *Penicillium chrysogenum* grown on MEA.

Figure S5. Temporal emission patterns of mVOCs originating from *Aspergillus niger* grown on PVC wall paper.

Figure S6. Temporal emission patterns of mVOCs originating from *Penicillium chrysogenum* grown on PVC wall paper.

Figure S7. Temporal emission patterns of mVOCs originating from *Alternaria alternata* grown on silicone rubber.

Figure S8. Temporal emission patterns of mVOCs originating from *Penicillium chrysogenum* grown on silicone rubber.

S1. Determination of fungal concentrations

After collecting bio-aerosols samples from 11 residential environments for measuring fungal concentrations, we sent MEA, used for a fungal growth medium, to our larboratory and cultivated fungi for 5 – 7 days at a temperature of 28°C. We counted the number of colonies every 24 hours to estimate airborne fungal concentrations. Equations used for estimating the airborne fungal concentrations are as below.

$$\mathrm{V} = \frac{Q \times t}{10^3}$$

$$V_{25} = V \times \frac{T_{25}}{T}$$

$$\mathrm{C} = \frac{CFU}{V_{25}}$$

where, V: Volume of collected air [m^3]
Q: Flow rate when collecting air samples [liter/min]
t: Sampling duration [min]
V_{25}: Converted volume of collected air at a temperature of 25 °C [m^3]
T_{25}: Absolute temperature of 25 °C [= 298K]
CFU: Number of fungal colonies
C: Airborne fungal concentration [CFU/m^3]

S2. Analysis of samples obtained from active air samplers

After collecting air samples from the active air samplers, we desorbed analytes using thermal desorption (Markes Unity Series 2, Markes International Ltd.) coupled with gas chromatography (Agilent 7820 A, Agilent Technology, USA) mass spectrometry (Agilent 5977 E, Agilent Technology, USA). We confirmed compounds using the Reference Standard Library (NT Search 2.0, National Institute of Standard and Technology, USA). For the quantitative analysis, we made calibration curves with the liquid standard adsorbed on the Tenax-TA tube with four different concentrations (i.e., 5, 10, 50, 500 ng/tube). In case that the quantification was not attainable for some compounds, we converted their concentrations with respect to benzene concentration. Correlation coefficients in the calibration curves were higher than 0.999 with all four concentrations (data not shown) and method detection limits ranged from 0.02 $\mu g/m^3$ (Xylene) to 0.25 $\mu g/m^3$ (Ethylbenzene).

S3. Analysis of samples obtained from passive air samplers

We analyzed the air samples obtained from passive air samplers using thermal desorption coupled with GC-MS under the same experimental conditions as the above section (S2).

Table S1. Characteristics of 11 residential environments recruited for our field experiments.

Household ID	Household characteristics			
	Types	House age (yr)	No. of residents	Note
R1	Apartment (7th floor)	17	4	Interior remodeling (summer)
R2	Apartment (1st floor)	17	4	Pet
R3	Apartment (5th floor)	25	4	-
R4	Apartment (5th floor)	23	4	-
R5	Apartment (2nd floor)	26	4	-
R6	Townhouse (4th floor)	20	4	-
R7	Apartment (5th floor)	18	4	Pet (winter only)
R8	Apartment (14th floor)	20	4	-
R9	Apartment (4th floor)	5	4	-
R10	Apartment (4th floor)	33	3	-
R11	Apartment (8th floor)	16	5	-

Table S2. Description of anlaysis conditions of thermal desorption coupled with gas chromatography/mass spectrometry (TD-GC-MS).

**Thermal desorption (UNITY2, Markes Internation, UK)**		
Desorption temperature		300°C for 10 min
Split ratio		20:1
Cold trap temperature		-10°C
**Gas chromatography (7820A, Agilent Technologies, USA)**		
Oven	Initial temperature	35°C
	Initial hold	5 min
	Ramp 1 temperature increase rate	10°C/min
	Ramp 1 temperature	220°C
	Ramp 1 hold	0 min
	Ramp 2 temperature increase rate	4°C/min
	Rampt temperature	280°C
	Ramp 2 hold	15 min
	Total running time	53.5 min
	Maximum temperature	320°C
	Equilibration time	0.2 min
Column	Model	DB-5
	Dimension	60 m length, 0.32 mm internal diameter, 1 μm film thickness
**Mass spectrometry (5977E, Agilent Technologies, USA)**		
Acquisition type		Scan mode
Tune type		Electron ionization
Start mass		17
End mass		350
Threshold		150

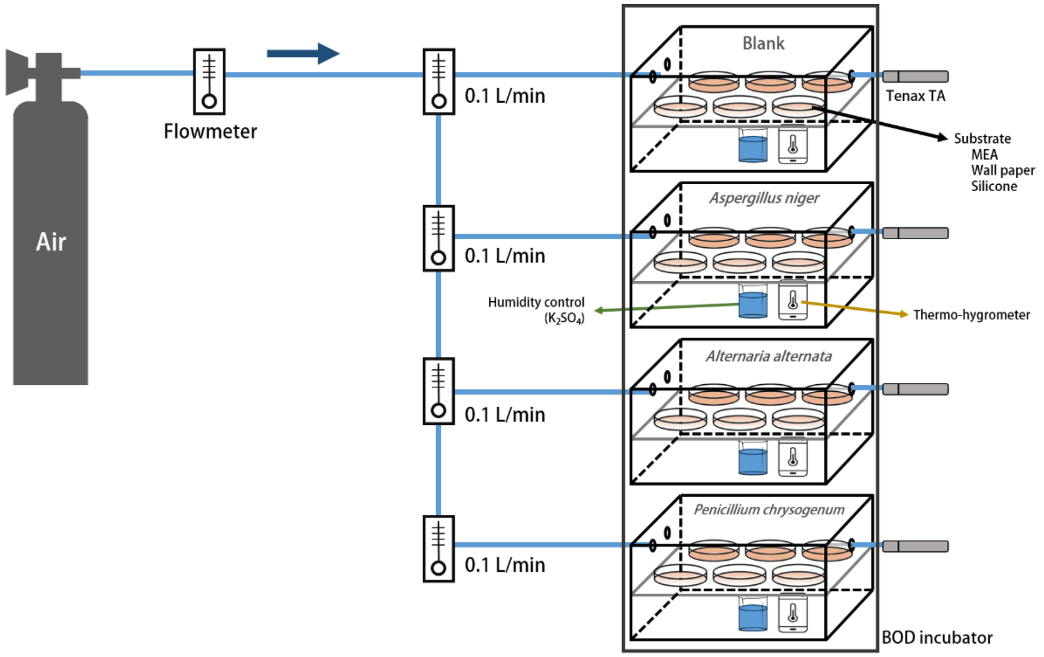

Figure S1. Schematic drawing of experimental setup for mVOCs emission testing.

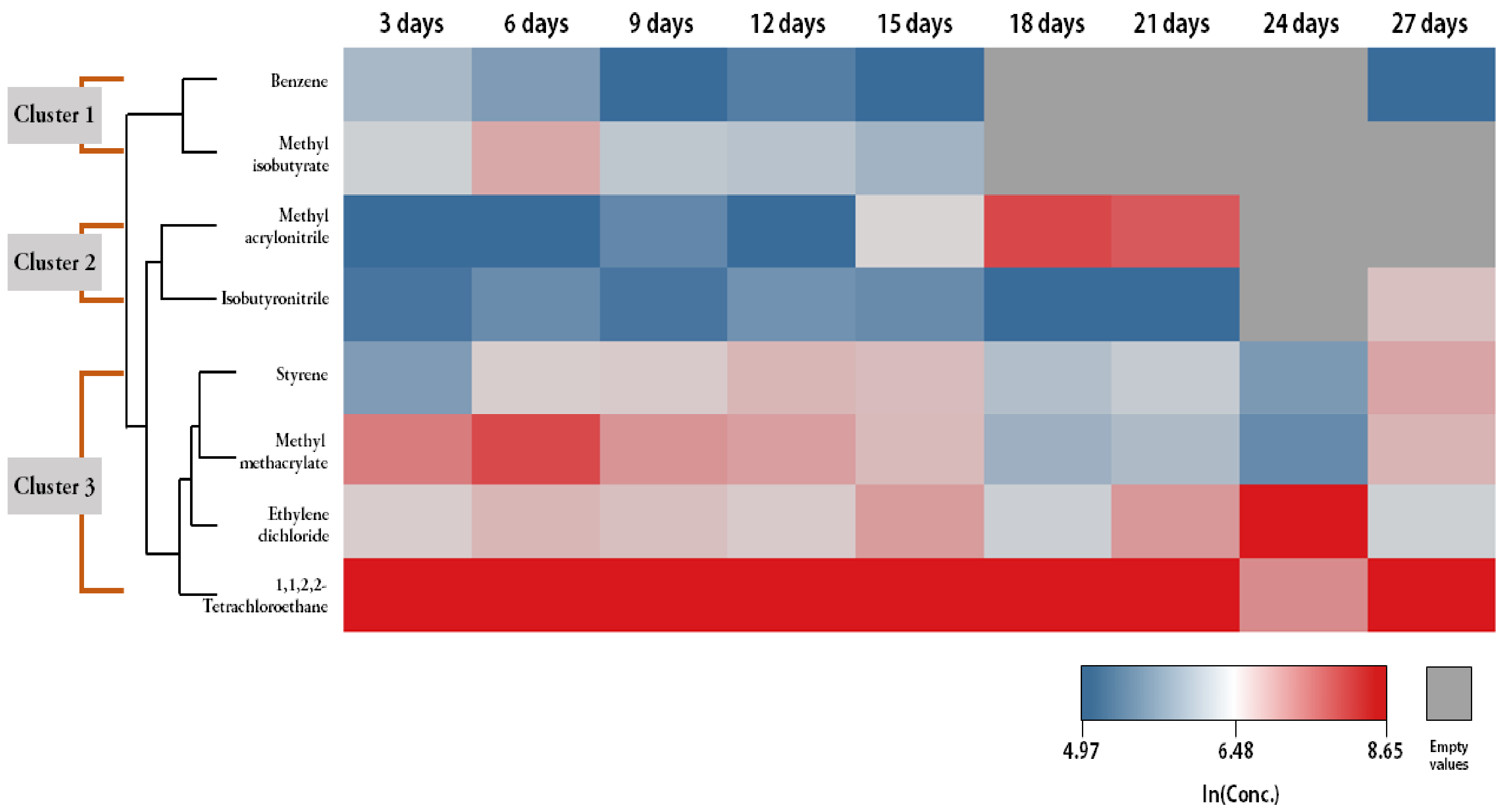

Figure S2. Temporal emission patterns of mVOCs originating from *Aspergillus niger* grown on MEA during the cultivation period. Depending on the temporal emission patterns, compounds were classified into three clusters.

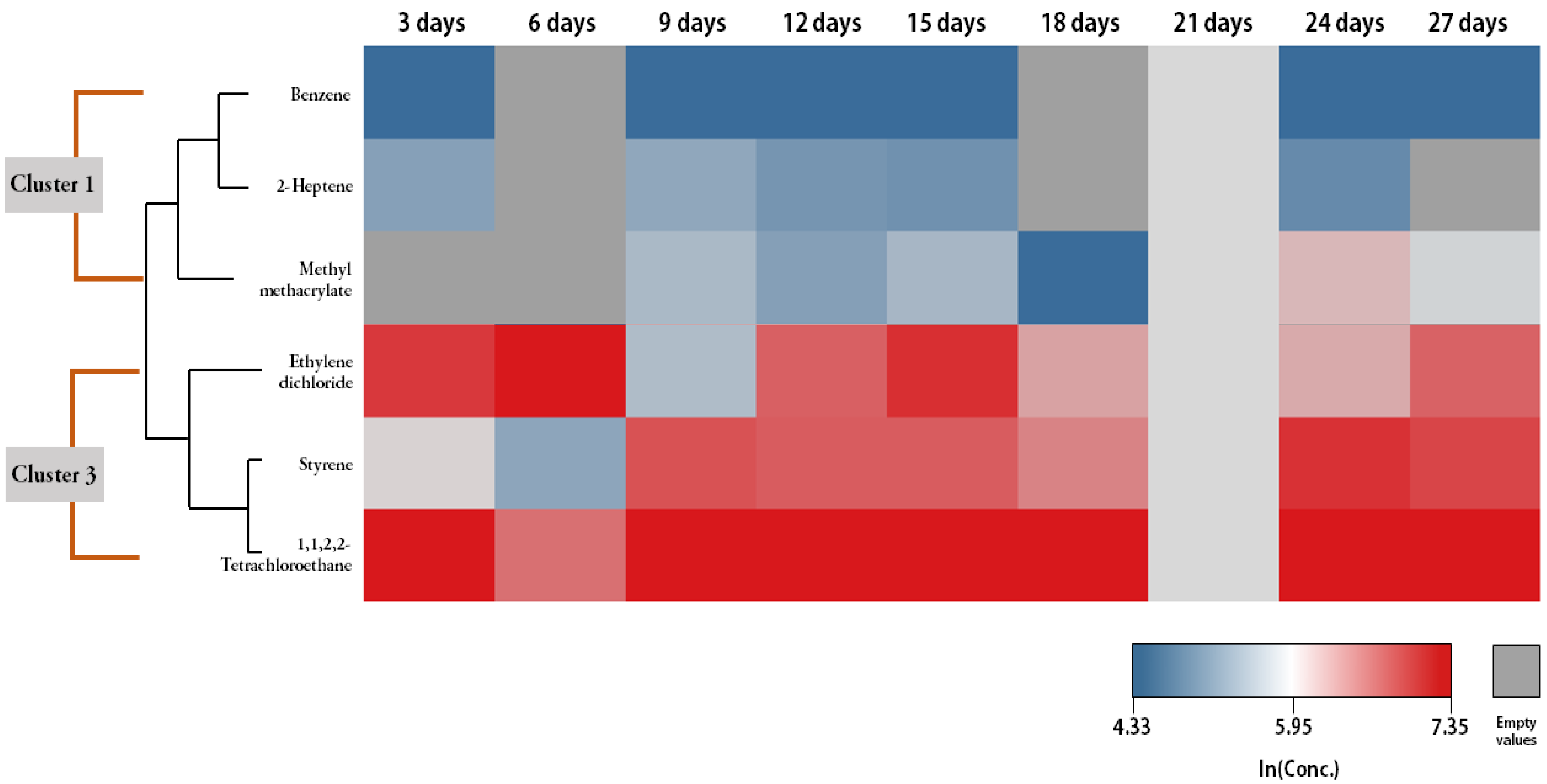

Figure S3. Temporal emission patterns of mVOCs originating from *Alternaria alternata* grown on MEA during the cultivation period. Depending on the temporal emission patterns, compounds were classified into two clusters.

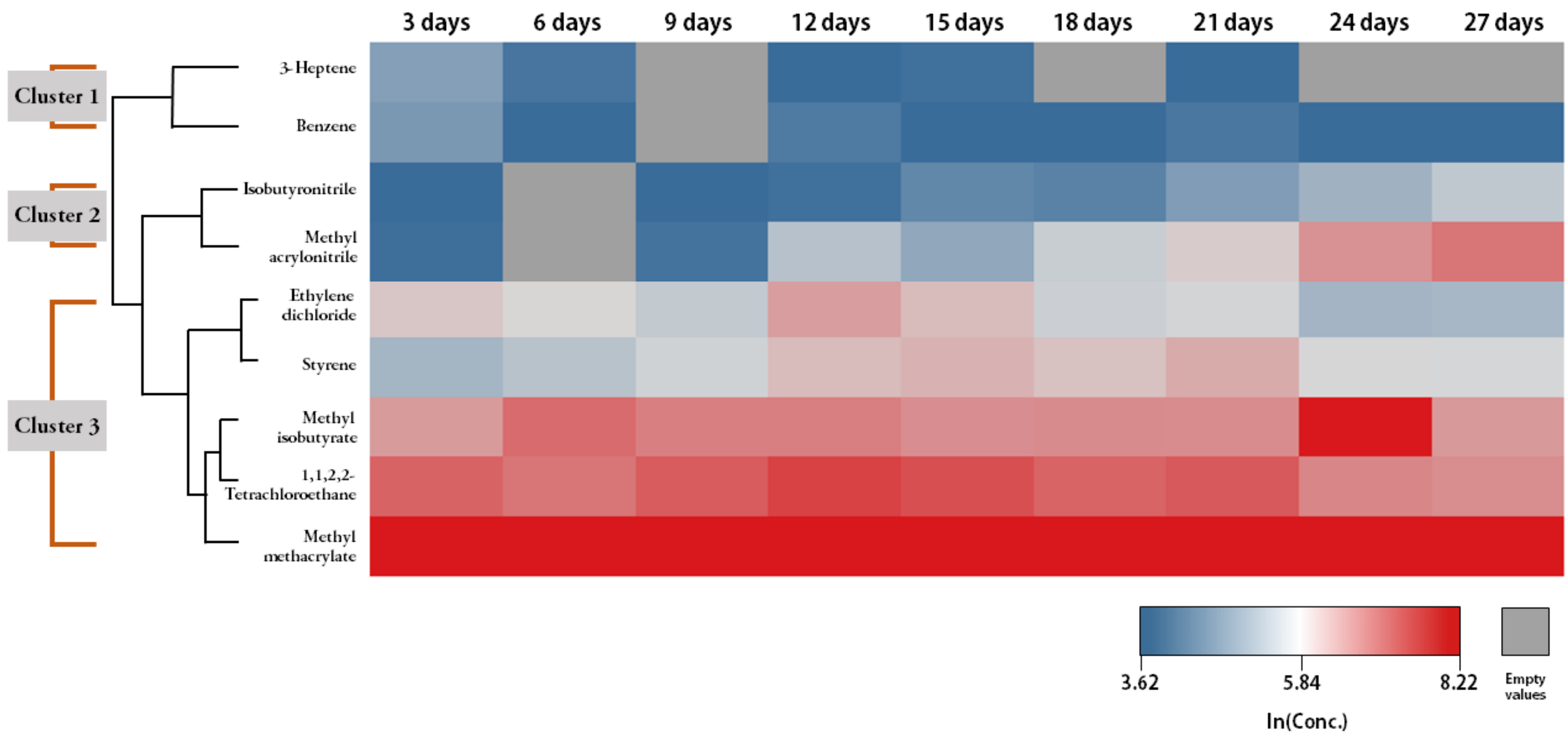

Figure S4. Temporal emission patterns of mVOCs originating from *Penicillium chrysogenum* grown on MEA during the cultivation period. Depending on the temporal emission patterns, compounds were classified into three clusters.

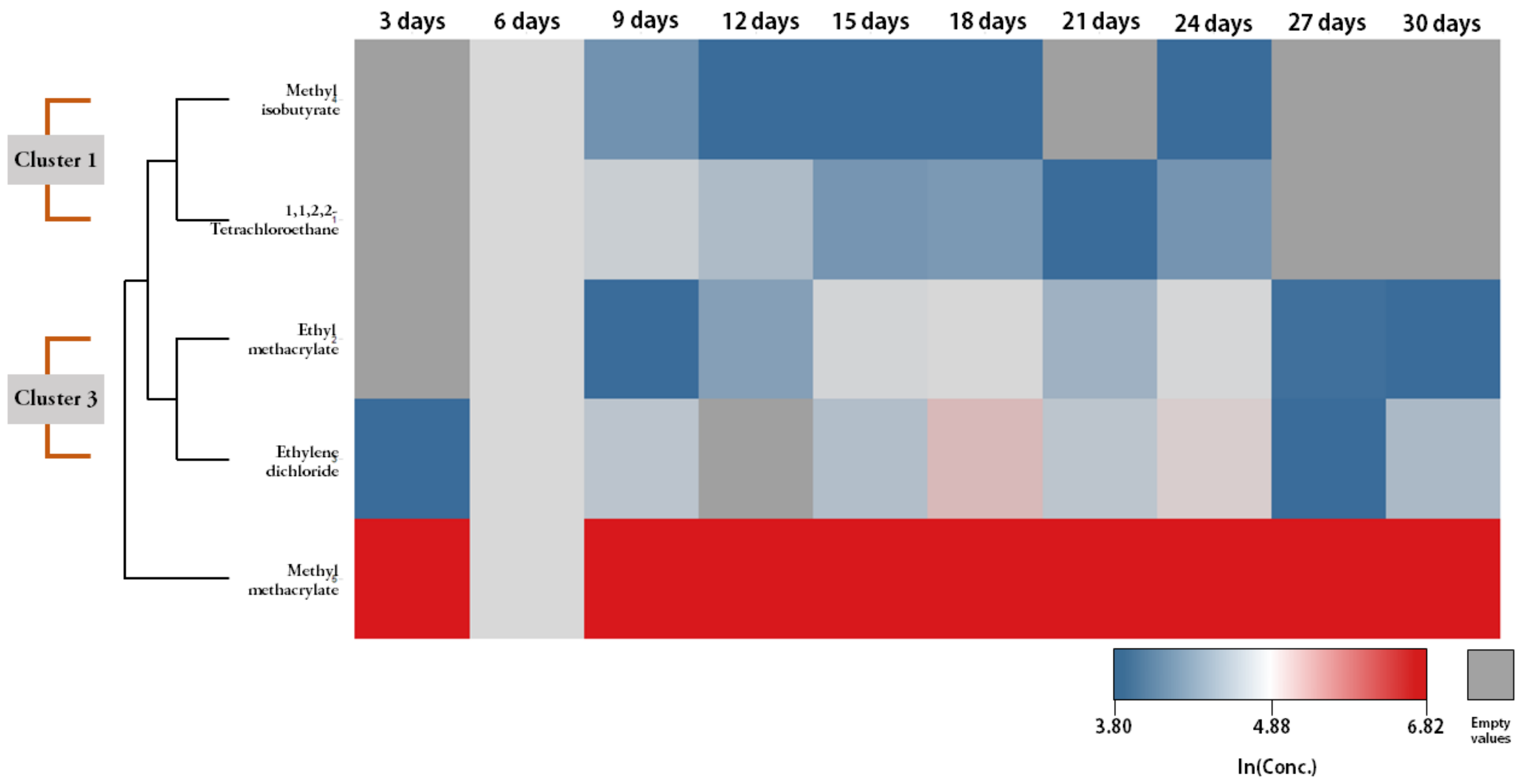

Figure S5. Temporal emission patterns of mVOCs originating from *Aspergillus niger* grown on PVC wall paper during the cultivation period. Depending on the temporal emission patterns, compounds were classified into two clusters.

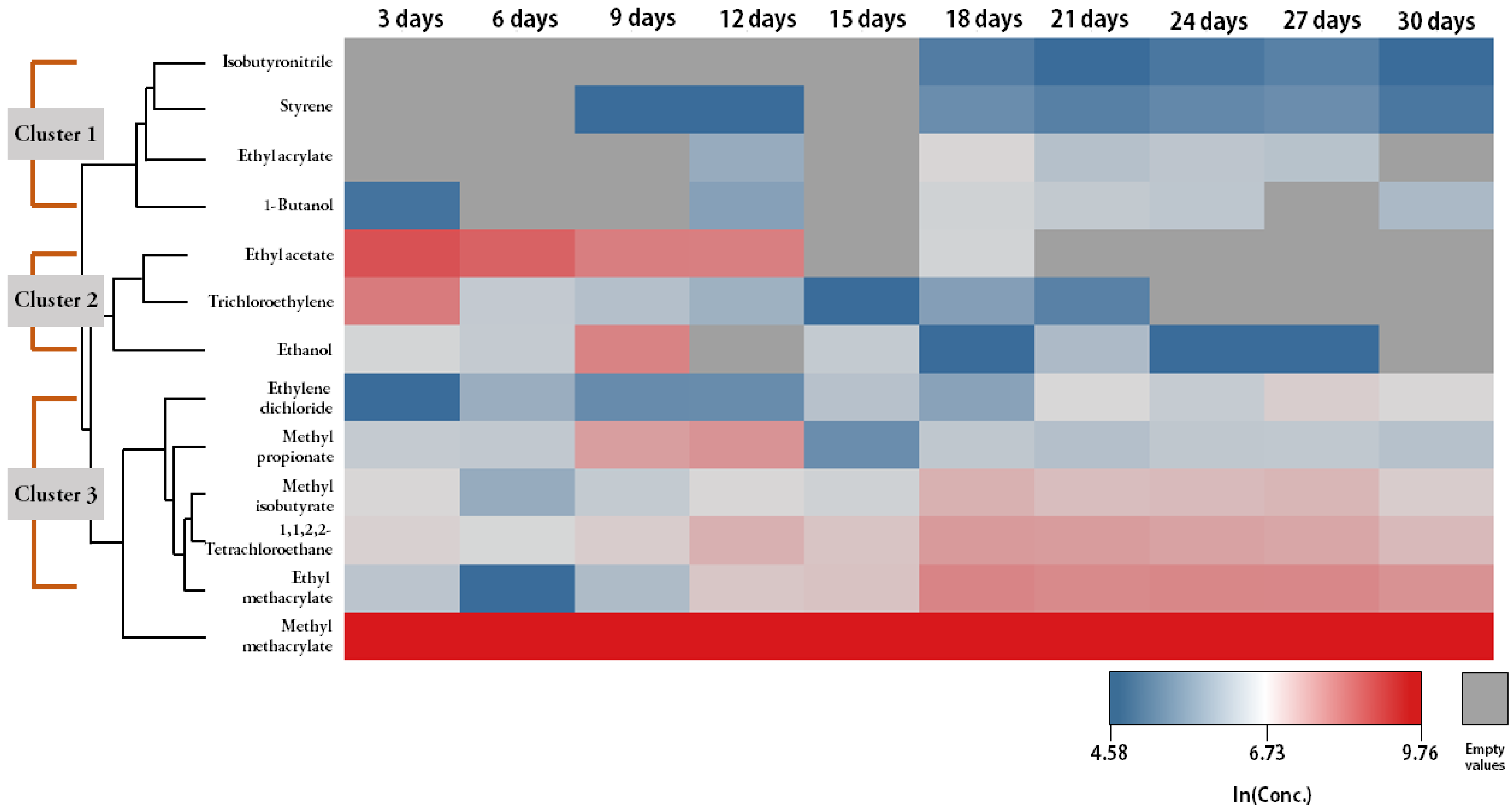

Figure S6. Temporal emission patterns of mVOCs originating from *Penicillium chrysogenum* grown on PVC wall paper during the cultivation period. Depending on the temporal emission patterns, compounds were classified into three clusters.

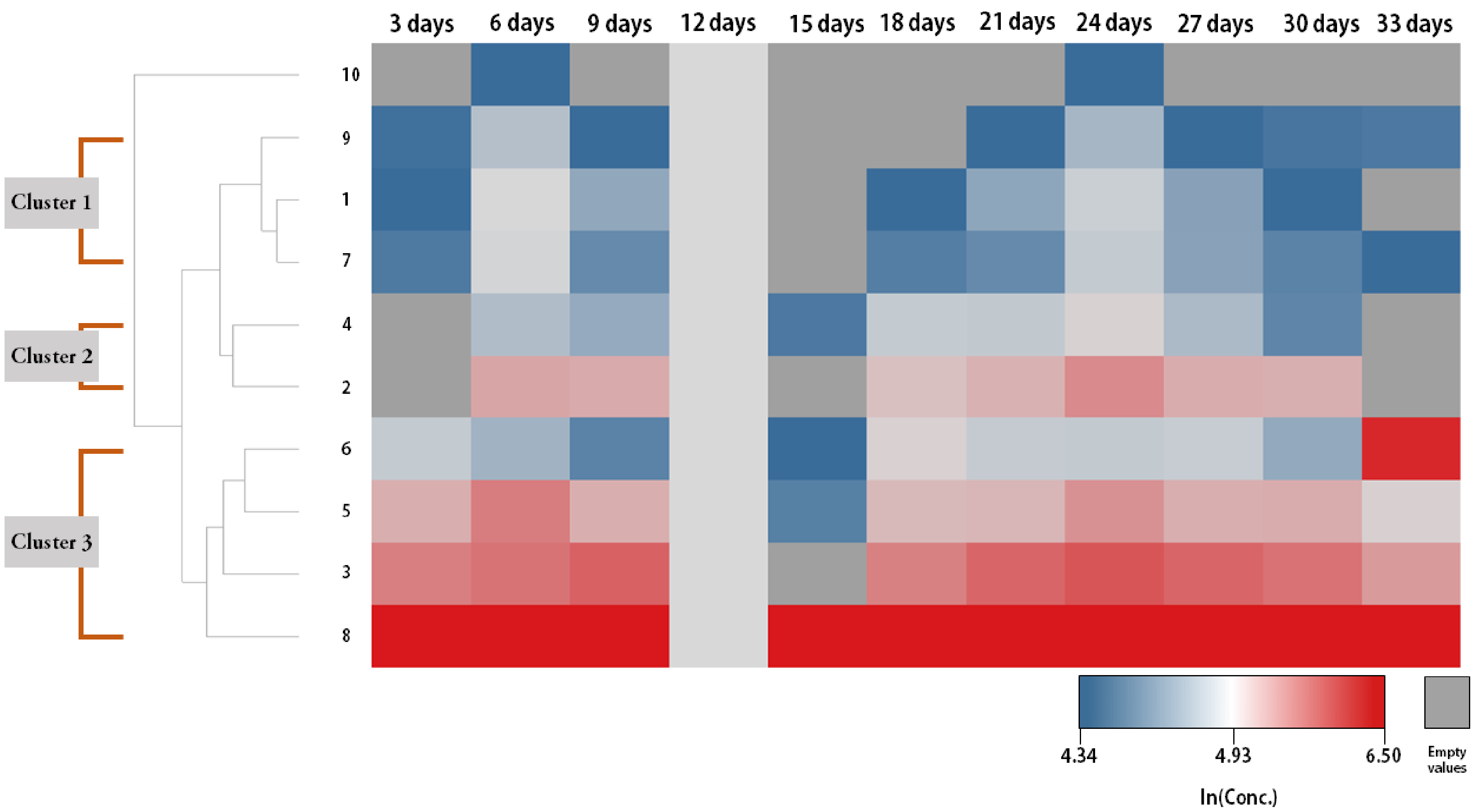

Figure S7. Temporal emission patterns of mVOCs originating from *Alternaria alternata* grown on silicone rubber during the cultivation period. Depending on the temporal emission patterns, compounds were classified into three clusters. Compounds were symbolized with numbers in the y-axis (See Table S5 for detailed information).

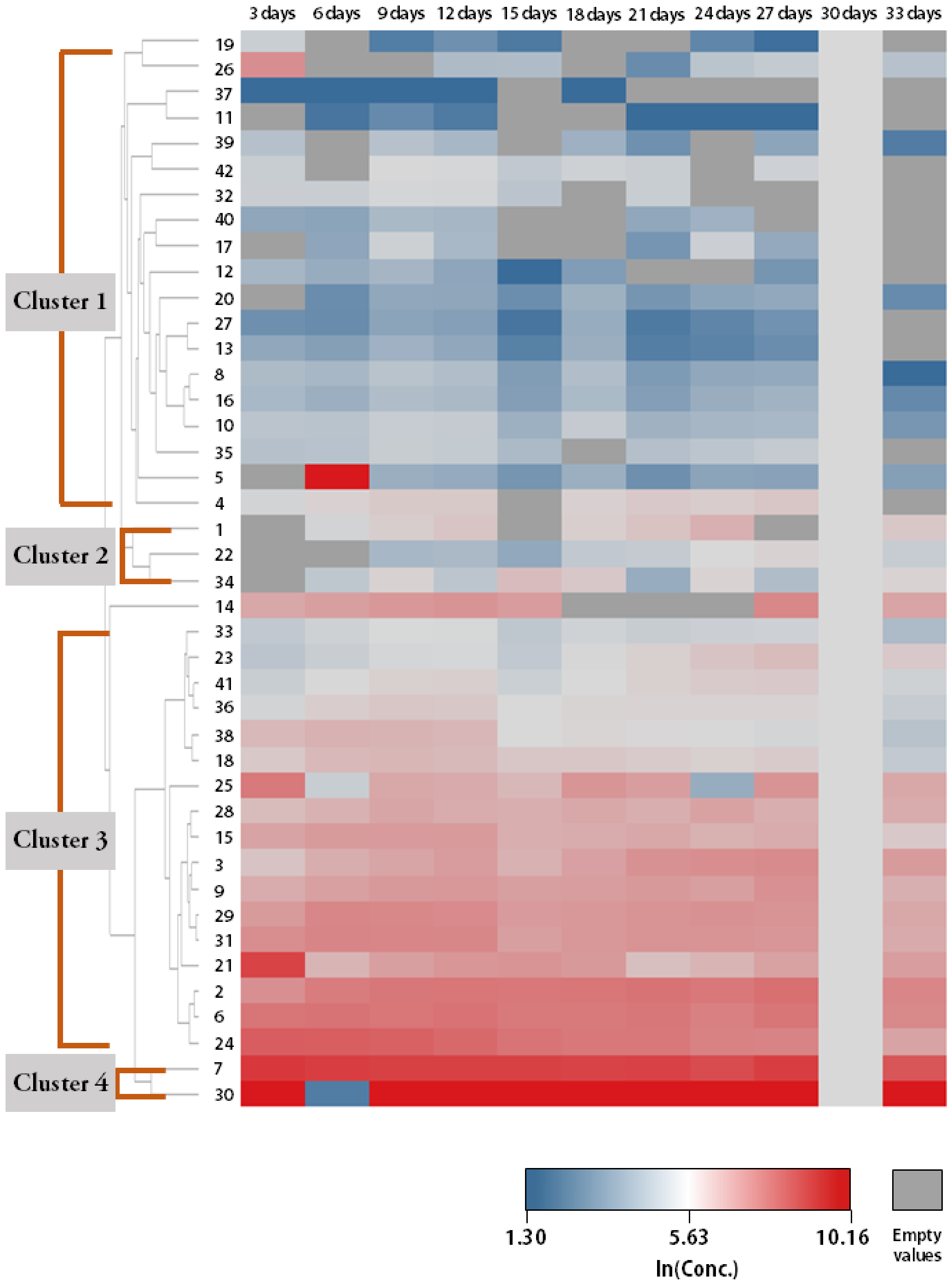

Figure S8. Temporal emission patterns of mVOCs originating from *Penicillium chrysogenum* grown on silicone rubber during the cultivation period. Depending on the temporal emission patterns, compounds were classified into four clusters. Compounds were symbolized with numbers in the y-axis (See Table S5 for detailed information).
